# Explainable artificial intelligence in dental imaging: a systematic review of interpretability and the current state of trust evidence

**DOI:** 10.3389/fdmed.2026.1948676

**Published:** 2026-09-11

**Authors:** Wlla E. Al-Hammad, Mohammad Y. Khatatbeh, Ghaida AlJamal, Mohammed Q. Shatnawi, Bara'a Fawaeer, Salem Alhatamleh, Abdel Rahman F. Shawaqfeh, Haron El-Rasheed Nijim, Muneer A. Al-Refai, Ahmad F. Hindawi, Anas Odat, Shams Eldin M. Saleh, Abdel Rahman M. Alzoubi, Khaled W. Tashtoush, Ahmad M. Alkhamaiseh, Loay E. Al-Hammad

**Affiliations:** 1 Department of Oral Medicine and Oral Surgery, Faculty of Dentistry, Jordan University of Science and Technology, Irbid, Jordan; 2 Department of Computer Information Systems, Faculty of Computer and Information Technology, Jordan University of Science and Technology, Irbid, Jordan; 3 Department of Medical Laboratory Sciences, Faculty of Applied Medical Sciences, Jordan University of Science and Technology, Irbid, Jordan; 4 Department of Computer Science, Faculty of Information Technology and Computer Sciences, Yarmouk University, Irbid, Jordan; 5 Faculty of Dentistry, Jordan University of Science and Technology, Irbid, Jordan

**Keywords:** clinical trust, dental imaging, explainable artificial intelligence, interpretability, risk of bias, systematic review

## Abstract

Explainable artificial intelligence (XAI) is increasingly used to improve the transparency of artificial intelligence models in dental and maxillofacial imaging, but the rigor with which explanations are evaluated remains unclear. This systematic review identified and categorized XAI approaches, their evaluation methods, and reported outcomes related to clinician trust and usability. The review was conducted in accordance with the PRISMA 2020 statement. Six electronic sources were searched, supplemented by Google Scholar and reference-list screening. Data were extracted on imaging modality, clinical application, explanation method, explanation scope, relationship to the predictive model, output format, evaluation approach, trust- or usability-related outcomes and external validation status. Risk of bias and applicability were assessed using QUADAS-2 and PROBAST. In total, 61 papers that met the specified inclusion criteria were identified. CAM-based local explanations predominated, whereas formal assessment of faithfulness, clinician trust, and usability was uncommon. Most included studies were judged to have a high overall risk of bias. Current evidence describes the technical use of XAI more strongly than its clinical utility. Future studies should use prespecified explanation taxonomies, quantitative faithfulness and localization tests, and clinician-centered evaluations of decision performance and calibrated reliance.

**Systematic Review Registration:**

https://www.crd.york.ac.uk/PROSPERO/view/CRD420261421868.

## 1 Introduction

### 1.1 Overview of artificial intelligence applications in dentistry and dental imaging

Artificial intelligence (AI) methods are increasingly investigated and applied in healthcare for tasks involving structured clinical data and medical imaging analysis (1). In dental imaging, machine learning (ML) methods, particularly deep learning (DL) models, have been applied to image classification, object detection, and segmentation in support of diagnostic applications (1, 2). These methods are well suited to image-analysis tasks involving complex visual patterns, as diagnostic image interpretation relies partly on visual pattern recognition and may be affected by interobserver variability.

AI has been investigated across multiple dental specialties, with imaging constituting a major area of research (3). Dental radiographs, intraoral photographs, and cone-beam computed tomography (CBCT) volumes can serve as inputs for classification, object-detection, segmentation, and clinical prediction models (3, 4). DL is widely used in these applications because it can learn hierarchical representations directly from image data during training (1). This capability supports tasks requiring the localization or delineation of findings such as caries, periapical lesions, periodontal bone loss, and jaw lesions. However, the available dental AI literature is frequently characterized by small or single-center datasets, variation in annotation procedures and imaging protocols, and limited external and clinical validation (2, 5). Strong performance on retrospective internal test datasets does not establish generalizability or clinical utility in routine practice. Assessment should therefore extend beyond task-specific model performance to include generalizability, clinical utility, and, where relevant, transparency and interpretability. When incorporated into clinical workflows, AI outputs may influence image interpretation, referral decisions, treatment planning, and communication with patients.

### 1.2 XAI in medical and dental imaging

The increasing use of complex DL models has increased interest in explainability in medical and dental imaging. Although DL models may achieve strong task-specific performance, the relationships between input features and model outputs are often not transparent to clinicians. This opacity may hinder clinical evaluation and acceptance, particularly where model-supported decisions may affect patient care and professional accountability (6).

Explainable AI (XAI) encompasses methods and design approaches intended to make model behavior or outputs more understandable to human users. Although explainability and interpretability are often used interchangeably, no universally accepted distinction exists between them. For this review, interpretability referred to the extent to which the relationships represented by a model could be understood directly, whereas explainability referred to methods that provide understandable information about the basis of a model output, particularly when the underlying model is complex or opaque (7). Common *post hoc* approaches in imaging include gradient-weighted class activation mapping (Grad-CAM) and other attribution-based visualization methods (8). Local interpretable model-agnostic explanations (LIME) approximate model behavior around an individual prediction using a local surrogate model, whereas Shapley additive explanations (SHAP) assign feature contributions using Shapley-value principles (9, 10). In imaging applications, the resulting attributions may be represented at the pixel, voxel, feature, or segmented-region level, depending on the implementation.

Explanation methods are relevant to dental imaging because a model may produce a correct output while relying on irrelevant anatomy, imaging artefacts, acquisition markers, or other spurious cues. For example, a model trained to detect caries or periapical lesions may achieve strong task-specific performance while relying on image characteristics that are not clinically relevant to the target finding. A visually plausible heatmap may therefore create an impression of credibility without establishing that the highlighted regions materially influenced the model output or that the model relies on clinically relevant evidence. Saliency-based explanations should consequently be evaluated using complementary assessments of localization, robustness, parameter sensitivity, faithfulness, and clinical relevance (11–13). The presence of an explanation alone does not establish its faithfulness, clinical relevance, or utility. Studies that present heatmaps only as illustrative outputs cannot determine whether those explanations correspond to expert-annotated regions, improve clinician diagnostic performance or understanding, or affect confidence and reliance (6, 11, 12).

Rigorous XAI evaluation may include expert review, quantitative localization against reference annotations, robustness testing, faithfulness assessment, usability evaluation, and reader studies examining clinician performance, confidence, reliance, or decision change. Faithfulness concerns whether an explanation corresponds to the model's predictive behavior rather than only producing a visually or clinically plausible output. Heatmaps that appear anatomically reasonable may not identify the input information that materially influenced the model output and should therefore be evaluated using complementary quantitative and human-centered methods (11–14). Without such evaluation, XAI outputs may function primarily as illustrative visualizations rather than validated explanations of model behavior or evidence of clinical utility (6).

### 1.3 Clinical trust, transparency, and ethical challenges

Clinician trust in and reliance on AI systems may be influenced by perceived performance, transparency, usability, prior experience, and the clinical context. These concepts should be distinguished: trust reflects a clinician's attitude toward a system, whereas reliance concerns the extent to which its outputs influence clinical decisions. For AI systems in dental imaging to provide clinical value, they should support a clearly defined clinical task, and any claimed improvement in decision-making should be demonstrated empirically. Their use should also preserve clinician oversight and professional accountability (1). Appropriate implementation requires clear reporting of the system's intended purpose, performance characteristics, limitations, and known failure modes (15).

Ethical implementation should address accountability, equity, privacy and data protection, explainability where appropriate, and clinician oversight (Cunha-Cruz et al., 2026). The role of an AI system should be defined in relation to clinician judgment and responsibility rather than assumed to replace them. Explainability also has ethical relevance because clinicians may require understandable information about the system's role, outputs, limitations, and uncertainty in diagnosis or treatment planning. Similar information may be relevant to patients when AI outputs are communicated as part of their care (15).

Recent healthcare AI reporting guidelines include the Transparent Reporting of a Multivariable Prediction Model for Individual Prognosis or Diagnosis–Artificial Intelligence (TRIPOD + AI) statement, the Checklist for Artificial Intelligence in Medical Imaging (CLAIM), and the Developmental and Exploratory Clinical Investigations of Decision-support Systems Driven by Artificial Intelligence (DECIDE-AI) guideline. Although these guidelines address different stages and types of AI evaluation, they collectively support transparent reporting of model development, validation, intended use, and, where applicable, early clinical evaluation (16–18). They also encourage clear description of how an AI system is intended to function within the relevant clinical pathway. Although these guidelines are not specific to XAI in dental and maxillofacial imaging, they support evaluation beyond task-specific predictive performance alone.

Previous reviews of dental AI have largely focused on applications, model performance, datasets, and model architectures (3–5, 19). While these reviews partially overlap with the scope of the present study, further focused evaluation of XAI specifically within dental and maxillofacial imaging is warranted, particularly regarding how generated explanations are evaluated and whether their clinical relevance has been assessed. Accordingly, this review aimed to: (1) identify and categorize XAI methods applied to dental and maxillofacial imaging; (2) characterize technical and human-centered evaluation practices, including assessments of faithfulness, localization, robustness, and expert review; and (3) assess reported outcomes related to clinician trust, reliance, and usability.

## 2 Materials and methods

### 2.1 Review question and objectives

This systematic review aimed to identify and categorize original studies that used XAI methods or intrinsic AI approaches in dental and maxillofacial imaging and to critically evaluate their methodological and clinical assessment practices. The specific objectives were to determine: (1) how interpretability or explanation mechanisms were implemented; (2) how explanations were evaluated using technical or human-centered methods; and (3) whether outcomes related to clinician trust, reliance, or usability were assessed.

### 2.2 Study design and registration

This systematic review was conducted and reported in accordance with the Preferred Reporting Items for Systematic Reviews and Meta-Analyses (PRISMA 2020) statement (20), and the protocol was registered in PROSPERO (ID: CRD420261421868)

### 2.3 Search strategy and protocol

A comprehensive search was conducted across six electronic sources: PubMed/MEDLINE, Scopus, Web of Science, IEEE Xplore, ScienceDirect, and SpringerLink, and additional records were identified through Google Scholar. The searches were conducted between April 2026 and May 2026. The search strategy was developed using various combinations of keywords with the Boolean operators “OR” and “AND”. Table 1 shows the search terms related to three concept groups. The search syntax was adapted to each database's requirements, and the complete database-specific search strategies are provided in Supplementary Table S1. Reference lists of the included studies were also manually screened to identify potentially eligible studies.

**Table 1:** Search terms grouped by concept.

Concept group	Search terms
Dental and maxillofacial imaging	“dental imaging”, “dental radiograph”, “panoramic”, “periapical”, “bitewing”, “CBCT”, “cephalometric”, “intraoral image”, “oral photograph”
Artificial intelligence	“artificial intelligence”, “machine learning”, “deep learning”, “CNN”, “transformer”, “neural network”
Explainability	“explainable artificial intelligence”, “explainable AI”, “interpretable”, “interpretability”, “explainable”, “explainability”, “Grad-CAM”, “SHAP”, “LIME”, “saliency map”, “attention map”, “occlusion”, “counterfactual”

The PIO scheme was utilised to evaluate the inclusion criteria as follows:

- Population: original studies using dental or maxillofacial imaging.
- Intervention: XAI methods applied to those images.
- Outcome: explanation generation type, evaluation method, and reported clinical trust/usability metrics.

### 2.4 Eligibility criteria

The eligibility criteria were limited to original research articles that involved human dental or maxillofacial imaging data, applied AI, ML, or DL methods, included an explicit explainability or interpretability component, were published in English, and reported sufficient methodological information for data extraction. Eligible explainability or interpretability components included, but were not limited to, Grad-CAM, CAM-based methods, saliency maps, attention maps, LIME, SHAP, occlusion sensitivity, feature-importance analysis, interpretable model architectures, or other transparent explanation outputs.

Review articles, systematic reviews, scoping reviews, meta-analyses, editorials, letters, conference abstracts without full papers, studies not related to dental or maxillofacial imaging, studies using AI without any explainability or interpretability method, non-English publications, animal-only studies, and purely simulation-based studies without clinical dental relevance were excluded.

### 2.5 Study selection

Two reviewers (HEN and ARFS) screened the titles and abstracts of all records retrieved from these initial search criteria. Disagreements were resolved by consulting a third reviewer (ARMA). If the abstract did not provide sufficient information to determine eligibility, or if the title appeared relevant but no abstract was available, the paper was selected for a full text review conducted by another two independent reviewers (MQS and AO). Subsequently, full-text articles that met the eligibility criteria were identified and included in the review.

### 2.6 Data extraction

Two independent reviewers (HEN and ARFS) used a standardized data extraction form to collect relevant information from each included study. Discrepancies in data extraction were resolved through discussion and consensus between the two reviewers. The following data were extracted:

1. Bibliographic information (author, year, country). Country was classified primarily according to the geographic source of the dataset or images; when this was not reported, author affiliation was used.
2. Dental imaging modality; multiple modalities were recorded when applicable.
3. Dental specialty and clinical application; when more than one clinical application was addressed, the principal clinical application of the study was used for classification.
4. XAI method used and the generated outputs
5. Type of explanation (local vs. global; *post-hoc* vs. intrinsic)
6. Evaluation approach for generated explanations.
7. Outcomes related to clinical trust, usability, interpretability, clinical confidence, decision support, or reported outcomes related to clinical adoption
8. External validation status (whether the model was tested on an independent dataset)

The full operational definitions for each extracted variable are provided in Supplementary Table S2.

### 2.7 Risk of bias assessment

Two reviewers (BF and AFH) independently assessed the risk of bias using two complementary tools: Quality Assessment of Diagnostic Accuracy Studies 2 (QUADAS-2) (21) and Prediction Model Risk of Bias Assessment Tool (PROBAST) (22). Of the 61 included studies, QUADAS-2 was applied to the 55 studies primarily focused on diagnostic accuracy and assessed four domains: patient selection, index test, reference standard, and flow and timing, together with applicability concerns. PROBAST was applied to the remaining six prediction-model studies and assessed risks related to participants, predictors, outcomes, and analysis. Domain-level and overall judgments were classified as low, high, or unclear risk of bias. Disagreements were resolved through discussion until consensus was reached. Cohen's kappa statistic was calculated to assess inter-rater reliability between the two reviewers for the QUADAS-2 and PROBAST risk-of-bias assessments.

## 3 Results

### 3.1 Study selection

**Figure 1:**
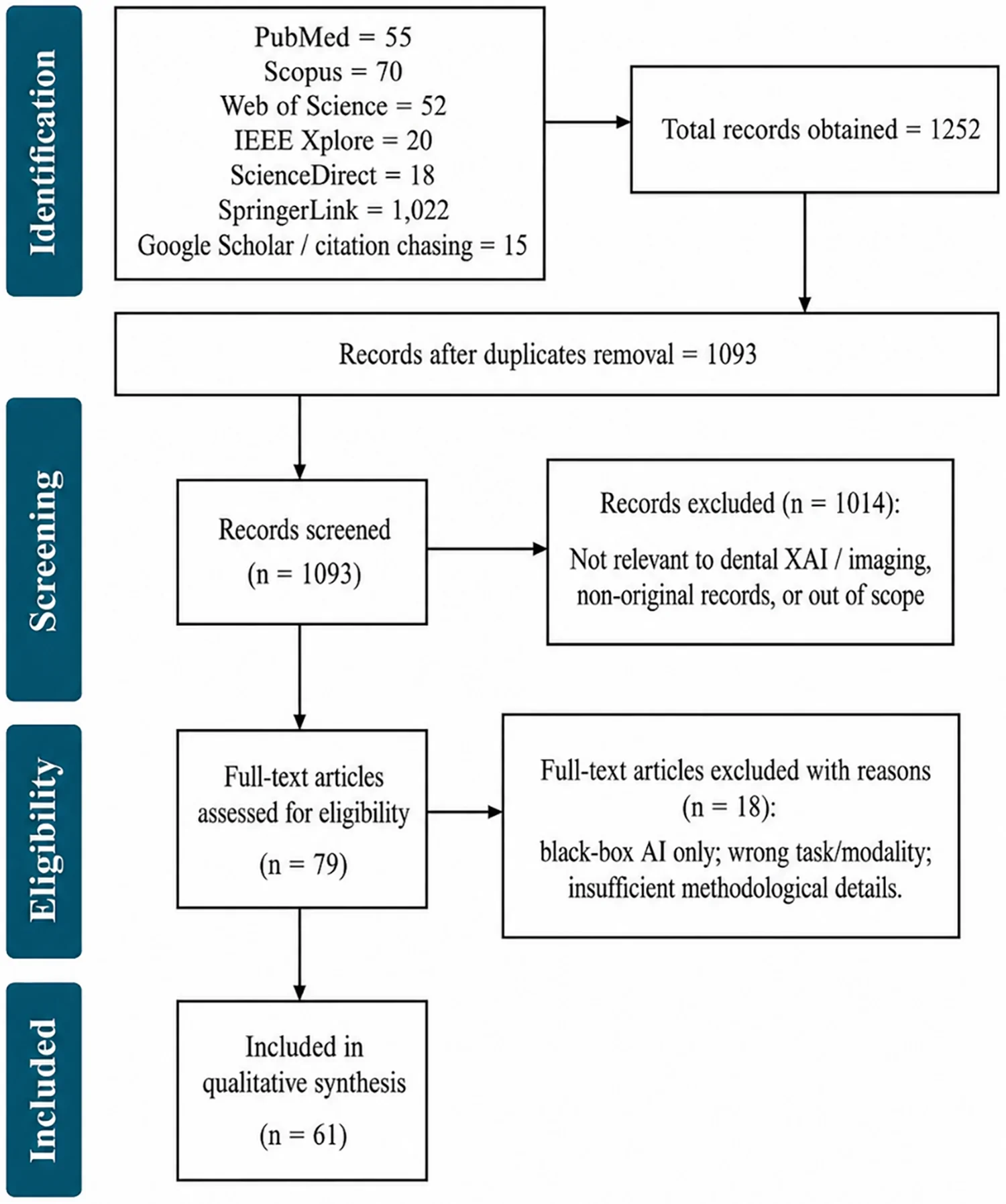
PRISMA flow diagram of study selection.

This systematic review followed the PRISMA guidelines, as shown in Figure. 1. Six databases were searched (PubMed, Scopus, Web of Science, IEEE Xplore, ScienceDirect, and SpringerLink) and additional records were identified through Google Scholar and reference-list screening. 1252 records were retrieved. After deduplication, 1,093 records were screened at the title and abstract stage. Of these, 1,014 records were excluded because they did not meet the eligibility criteria, leaving 79 articles for full-text assessment. Following full-text review, 18 articles were excluded with documented reasons, and 61 studies met the inclusion criteria and were included in the final synthesis.

### 3.2 Characteristics of the included studies

A summary of the extracted data from the 61 included studies is presented in Table 2. Extracted variables included bibliographic information, imaging modality, dental application, XAI method, explanation scope, model dependency, and evaluation type. The complete extraction table with all variables is available in Supplementary Table S3.

**Table 2:** Summary of included studies with condensed extraction (*n* = 61).

#	Author & year*	Imaging modality	Dental application	XAI method	Scope	Model dependency	Evaluation type
1	Asiri, 2025 (32)	Intraoral photographs	Dental caries	Grad-CAM	Local	Post-hoc	Qualitative
2	Sreeram, 2025 (33)	Panoramic radiographs	OMFR/ OMFS	Grad-CAM	Local	Post-hoc	Qualitative
3	Çakmak, 2026 (34)	Panoramic radiographs	Endodontics	Grad-CAM	Local	Post-hoc	Qualitative
4	Ovuz, 2026 (35)	CBCT	OMFR/ OMFS	Grad-CAM	Local	Post-hoc	Qualitative
5	Palaz, 2026 (36)	Panoramic radiographs	Pediatric dentistry	Grad-CAM; SmoothGrad; Occlusion sensitivity	Local	Post-hoc	Expert
6	Ali, 2024 (37)	Intraoral radiographs	Forensic dentistry	SHAP	Global	Post-hoc	Feature-importance
7	Rana, 2026 (38)	Panoramic radiographs	OMFR/ OMFS	Grad-CAM; SHAP; Saliency maps	Local	Post-hoc	Qualitative
8	Oztekin, 2023 (39)	Panoramic radiographs	Dental caries	Grad-CAM	Local	Post-hoc	Localization
9	Bergner, 2022 (40)	Intraoral radiographs	Dental caries	Saliency maps; Grad-CAM; Occlusion sensitivity	Local	Intrinsic	Localization
10	Pishghadam, 2025 (41)	CBCT	Forensic dentistry	Grad-CAM; Attention maps	Local	Both	Expert
11	Cai, 2023 (42)	3D facial scans	Orthodontics	Grad-CAM	Local	Post-hoc	Quantitative
12	Kim, 2025 (43)	Intraoral photographs	Dental caries	Grad-CAM	Local	Post-hoc	Localization
13	Ashi, 2026 (44)	Intraoral photographs	Dental caries	Grad-CAM	Local	Post-hoc	Qualitative
14	Qi, 2026 (45)	CBCT	Dental caries	CAM	Local	Post-hoc	Qualitative
15	Asghar, 2025 (23)	Intraoral photographs	Dental caries	Grad-CAM	Local	Post-hoc	Qualitative
16	Jung, 2026 (27)	Panoramic radiographs	Orofacial pain	Grad-CAM	Local	Both	Ablation
17	Zhang, 2025 (46)	Panoramic radiographs	Periodontology	Grad-CAM	Local	Post-hoc	Qualitative
18	Hayashi-Sakai, 2025 (47)	Panoramic radiographs	Pediatric dentistry	SHAP	Local	Post-hoc	Qualitative
19	Xu, 2025 (48)	CBCT	Orthodontics	Grad-CAM	Local	Post-hoc	Expert
20	Ong, 2024 (49)	Panoramic radiographs	Forensic dentistry	Grad-CAM	Local	Post-hoc	Qualitative
21	Liu, 2024 (50)	Intraoral radiographs	Dental caries	Grad-CAM	Local	Post-hoc	Expert
22	Vivel-Couso, 2026 (51)	Panoramic radiographs	Forensic dentistry	Grad-CAM	Local	Both	Questionnaire
23	Barzas, 2025 (52)	CBCT	OMFR/ OMFS	Grad-CAM++	Local	Post-hoc	Qualitative
24	Chen, 2024 (53)	CBCT	Dental caries	LIME	Local	Post-hoc	Expert
25	Duru, 2026 (54)	Panoramic radiographs	Forensic dentistry	Integrated gradients	Local	Post-hoc	Expert
26	Inani, 2024 (55)	Intraoral photographs	Dental caries	Grad-CAM	Local	Post-hoc	Qualitative
27	Parathanath, 2025 (56)	Intraoral radiographs	Dental caries	Attention maps	Unclear	Intrinsic	Ablation
28	Tahmasbi, 2026 (57)	Extraoral photographs	Orthodontics	Saliency maps	Local	Post-hoc	Qualitative
29	Zhu, 2025 (58)	Panoramic radiographs	OMFR/ OMFS	Visual analytics	Both	Post-hoc	Usability
30	Gwak, 2024 (59)	Panoramic radiographs	OMFR/ OMFS	Grad-CAM	Local	Post-hoc	Localization
31	Arian, 2023 (24)	Panoramic radiographs	Forensic dentistry	FullGrad	Local	Post-hoc	Qualitative
32	Wen, 2024 (60)	Intraoral photographs	Periodontology	Grad-CAM++	Local	Post-hoc	Localization
33	Chen, 2023 (61)	Panoramic radiographs	Dental caries	Rule-based visual explanation	Local	Intrinsic	Quantitative
34	Kang, 2024 (62)	Panoramic radiographs	OMFR/ OMFS	Attention maps	Both	Intrinsic	Localization
35	Motmaen, 2024 (63)	Panoramic radiographs	OMFR/ OMFS	CAMERAS class activation mapping	Local	Post-hoc	Expert
36	Buyukcakir, 2025 (64)	CBCT	Forensic dentistry	Attention rollout	Both	Intrinsic	Qualitative
37	Lee, 2024 (65)	MRI	Orofacial pain	Grad-CAM	Local	Post-hoc	Quantitative
38	Yeom, 2023 (66)	Panoramic radiographs	Forensic dentistry	Attention rollout	Local	Post-hoc	Qualitative
39	Chen, 2025 (67)	CBCT	OMFR/ OMFS	Grad-CAM	Both	Post-hoc	Quantitative
40	Li, 2025 (68)	CBCT	Orthodontics	Integrated Gradients; SHAP; LIME; Attention maps	Both	Both	Expert
41	Zhang, 2023 (69)	Cephalometric radiographs	Orthodontics	Grad-CAM	Local	Post-hoc	Qualitative
42	Miranda, 2023 (70)	CBCT	Orthodontics	Grad-CAM; Attention maps	Local	Both	Expert
43	Chung, 2025 (71)	Intraoral photographs	Orthodontics	Score-CAM	Local	Post-hoc	Expert
44	Zhou, 2023 (72)	Intraoral photographs	Forensic dentistry	Grad-CAM	Local	Post-hoc	Qualitative
45	Simsek, 2025 (73)	Panoramic radiographs	Forensic dentistry	SHAP	Global	Post-hoc	Feature-importance
46	Lee, 2022 (74)	MRI	Orofacial pain	Grad-CAM	Local	Post-hoc	Quantitative
47	Kayadibi, 2025 (75)	Panoramic radiographs	OMFR/ OMFS	EigenCAM	Local	Post-hoc	Usability
48	Choi, 2023 (76)	Intraoral radiographs	Endodontics	CAM	Local	Post-hoc	Qualitative
49	Moreira, 2026 (77)	CBCT	Forensic dentistry	Grad-CAM	Local	Post-hoc	Qualitative
50	Lee, 2024 (25)	Panoramic radiographs; MRI	Orofacial pain	Grad-CAM	Local	Post-hoc	Qualitative
51	Pereira, 2025 (78)	Panoramic radiographs	Forensic dentistry	Grad-CAM	Local	Post-hoc	Qualitative
52	Buyukcakir, 2025 (79)	Panoramic radiographs	Forensic dentistry	Attention maps; Visual analytics	Both	Both	Qualitative
53	Joo, 2023 (80)	Panoramic radiographs	Forensic dentistry	Visual analytics	Both	Intrinsic	Quantitative
54	Qiu, 2026 (81)	CBCT	OMFR/ OMFS	SHAP	Both	Post-hoc	Feature-importance
55	Ragodos, 2022 (26)	Intraoral photographs	OMFR/ OMFS	Saliency maps	Local	Post-hoc	Qualitative
56	Zhang, 2022 (82)	Cephalometric radiographs	Orthodontics	Grad-CAM	Both	Post-hoc	Quantitative
57	Xu, 2024 (83)	CBCT	Orthodontics	Grad-CAM	Local	Post-hoc	Qualitative
58	Kavousinejad, 2026 (28)	Intraoral photographs	Orthodontics	Grad-CAM; Attention maps	Local	Both	Expert
59	Luo, 2026 (84)	Panoramic radiographs	Forensic dentistry	Grad-CAM; Attention maps	Local	Both	Quantitative
60	Sivari Resul, 2026 (85)	Panoramic radiographs	Pediatric dentistry	Grad-CAM; Attention maps	Local	Both	Expert
61	Khurshid, 2026 (86)	Intraoral photographs	Dental caries	Grad-CAM	Local	Post-hoc	Qualitative

Year*: studies assigned a 2026 publication year include articles available online ahead of their final issue publication at the time of the literature search. CBCT, cone-beam computed tomography; MRI, magnetic resonance imaging; OMFR/OMFS, oral and maxillofacial radiology/surgery; XAI, explainable AI; CAM, class activation mapping; Grad-CAM, gradient-weighted class activation mapping; SHATableCaptionP, shapley additive explanations; LIME, local interpretable model-agnostic explanations; LRP, layer-wise relevance propagation; EigenCAM, eigen decomposition-based class activation mapping; FullGrad, full-gradient representation for neural network visualization; CAMERAS, class activation mapping for three-dimensional images.

The included studies were published from 2022 to 2026 and were associated with multiple countries. Based on the country-classification criterion specified in the Methods, China contributed the largest number of studies (*n* = 18, 29.5%), followed by South Korea (*n* = 11, 18.0%), Turkey (*n* = 8, 13.1%), India (*n* = 4, 6.6%), Iran (*n* = 3, 4.9%), and Brazil (*n* = 3, 4.9%). Two studies each were assigned to Saudi Arabia and Belgium (*n* = 2, 3.3% each), whereas the United States, Germany, Japan, Spain, Uruguay, Portugal, Indonesia, and Bangladesh contributed one study each (*n* = 1, 1.6%). For two studies (3.3%), the geographic origin of the imaging data was not reported because publicly available datasets were used (23, 24).

Panoramic radiography was the most frequently reported imaging modality (*n* = 25, 41.0%), followed by CBCT (*n* = 13, 21.3%), intraoral photographs (*n* = 11, 18.0%), and intraoral radiography, including bitewing and periapical imaging (*n* = 5, 8.2%). Other modalities included magnetic resonance imaging (MRI; *n* = 3, 4.9%), cephalometric radiography (*n* = 2, 3.3%), extraoral photography (*n* = 1, 1.6%), and three-dimensional facial scanning (*n* = 1, 1.6%). Reported dataset sizes varied considerably, but direct comparison was limited because the units of analysis differed among studies and included radiographs, photographs, CBCT volumes, and other image-derived units. The smallest reported dataset comprised 16 images (25), whereas the largest comprised 38,486 images (26).

The studies addressed several clinical domains and target applications. Forensic dental applications constituted the largest clinical application category (*n* = 15, 24.6%), followed by caries-related applications (*n* = 13, 21.3%), oral and maxillofacial applications currently grouped under radiology or surgery (*n* = 12, 19.7%), and orthodontic applications (*n* = 10, 16.4%). Less frequently represented categories included orofacial pain (*n* = 4, 6.6%), pediatric dentistry (*n* = 3, 4.9%), periodontology (*n* = 2, 3.3%), and endodontics (*n* = 2, 3.3%).

### 3.3 XAI methods and explanation outputs

A total of 76 XAI method instances were extracted from the included studies. Because some studies reported more than one explainability method, these counts represent XAI method instances rather than the number of individual studies. The most widely adopted XAI method was Grad-CAM (*n* = 36, 47.4%), followed by attention maps (*n* = 9, 11.8%) and SHAP (*n* = 6, 7.9%). CAM-family approaches constituted the most frequently represented method group. Non-CAM gradient-based attribution methods and attention-based explanation or visualization approaches were also reported. Because descriptors such as gradient-based, perturbation-based, model-agnostic, and framework-level represent different and potentially overlapping dimensions, their frequencies should be interpreted according to the classification rules specified in the Methods.

At the study level, local explanations were reported in 49 studies (80.3%). Nine studies (14.8%) reported both local and global explanations approaches, two studies (3.3%) reported global explanations only, and one study (1.6%) had unclear explanation scope. Regarding the relation between explanation approach and the predictive model 46 studies (75.4%) used *post-hoc* explanation methods, nine studies (14.8%) included both *post-hoc* and intrinsic approaches, and six studies (9.8%) used intrinsic approaches alone.

A total of 86 individual output instances were extracted from the included studies. Heatmap-based outputs were the most common (*n* = 45, 52.3%), including standard heatmaps, SHAP heatmaps, CAM overlay and CAM, 3D heatmaps, and 3D surface heatmaps. Attention-based outputs were the second most frequent (*n* = 9, 10.5%), comprising attention maps, attention-weighted feature maps, binarized attention maps, 3D attention maps, and segmentation maps derived from attention. Gradient-based outputs (*n* = 7, 8.1%) included saliency maps and activation maps. Other visual outputs appeared, followed by map variants, feature importance plots, segmentation-based outputs, overlay and mask outputs, and text and structured outputs. More details about the distribution of XAI outputs across included studies are available in Supplementary File S4.

### 3.4 Evaluation of XAI explanations

Qualitative assessment was the most frequently reported explanation-evaluation approach (*n* = 27, 44.3%), followed by expert or clinician review (*n* = 12, 19.7%) and quantitative evaluation (*n* = 8, 13.1%). The quantitative approaches included analyses of saliency distributions and explanation characteristics, latent-feature contributions and nomograms, and simulation-based evaluation. These quantitative assessments did not constitute formal faithfulness testing according to the operational definition used in this review. Formal localization-based evaluation, such as comparison with lesion masks, annotated regions, or overlap-based criteria, was reported in six studies (*n* = 6, 9.8%). Less frequent approaches included feature-importance analysis (*n* = 3, 4.9%), usability assessment and ablation-based evaluation (*n* = 2 each, 3.3%), and questionnaire-based was reported in one study (*n* = 1, 1.6%). More details about the distribution of XAI evaluation characteristics across included studies are available in Supplementary File S5.

At the study level, clinician or expert review of explanations was reported in 28 studies (45.9%), whereas 33 studies (54.1%) did not report formal clinician review of explanations. Reader studies, defined as structured evaluations involving human readers or clinicians assessing cases or model outputs, were reported in 16 studies (26.2%), while 45 studies (73.8%) did not include a reader-study component. A broad localization or anatomical plausibility check was documented in 56 studies (91.8%); however, most of these checks were qualitative rather than based on formal localization metrics. In contrast, none of the included studies (0%) reported evaluating explanation faithfulness.

Outcomes directly related to clinical trust and usability were infrequently reported. Trust was measured in only one study (*n* = 1, 1.6%), whereas 60 studies (98.4%) did not assess clinician trust in XAI-generated explanations. Usability was evaluated in three studies (*n* = 3, 4.9%), while 58 studies (95.1%) did not report usability assessment.

### 3.5 External validation

Two studies (*n* = 2, 3.3%) performed external validation of the AI model using an independent dataset from a different institution or geographic location (27, 28). The remaining 59 studies (96.7%) relied solely on internal validation using data splits from a single dataset.

### 3.6 Risk of bias and applicability assessment

**Figure 2:**
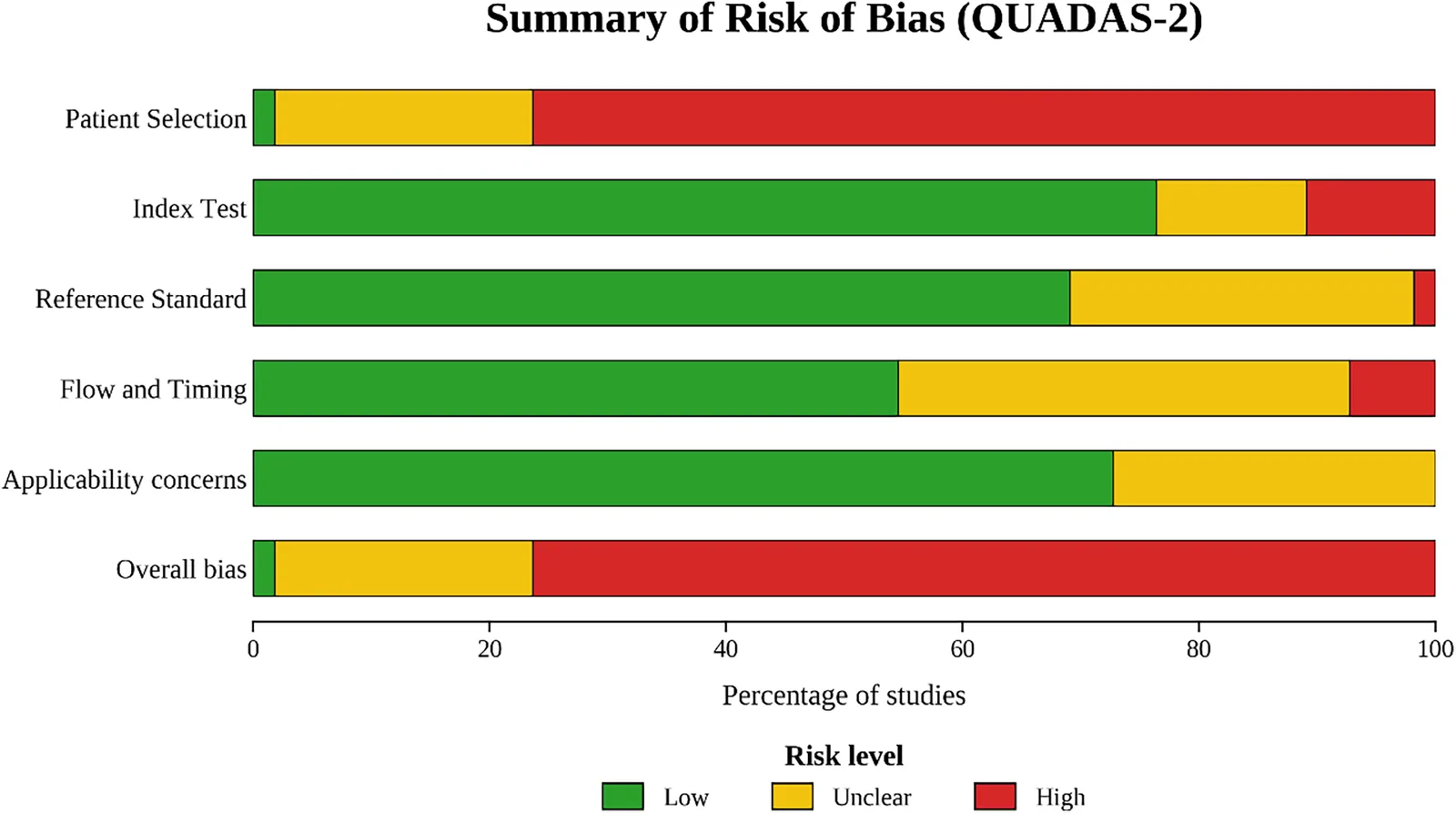
Risk-of-bias and applicability assessment using QUADAS-2.

**Figure 3:**
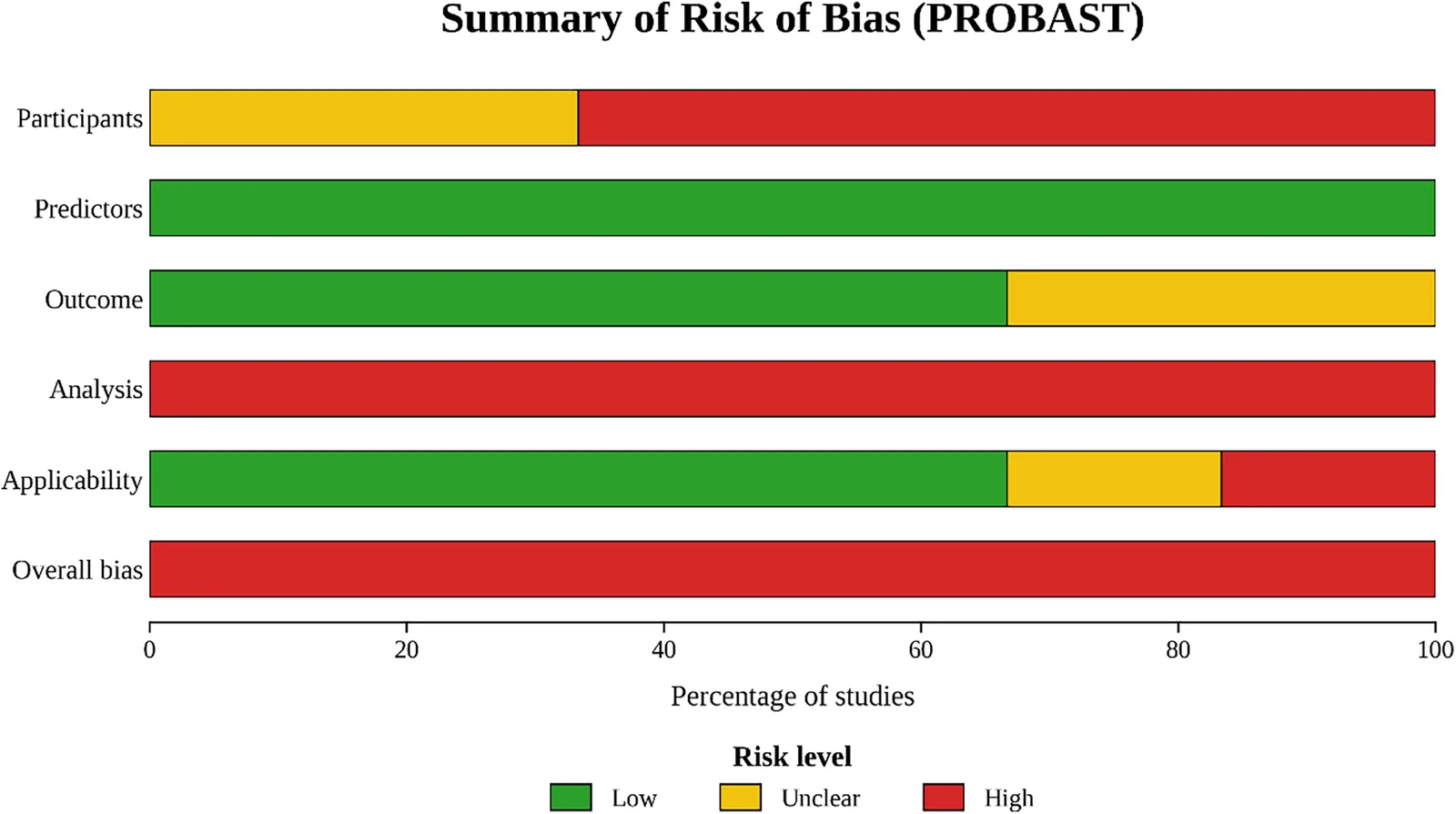
Risk-of-bias and applicability assessment using PROBAST.

Among the 55 studies assessed using QUADAS-2, 42 studies (76.4%) were judged to have a high overall risk of bias, one study (1.8%) was judged to have a low overall risk of bias, and 12 studies (21.8%) were judged to have an unclear overall risk of bias. Among the six studies assessed using PROBAST, all of them were judged to have a high overall risk of bias (100%). Overall, 48 studies (78.9%) had high overall risk of bias. Detailed domain-specific results are presented in Figure. 2 for QUADAS-2 and Figure. 3 for PROBAST. Inter-rater reliability yielded an average Cohen's kappa of *κ* = 0.7–1.0 across all domains, indicating substantial to perfect agreement.

## 4 Discussion and conclusions

This systematic review synthesized 61 unique reports on XAI in dental and maxillofacial imaging published between 2022 and 2026. The review specifically examined the XAI methods used, the approaches applied to evaluate explanations, and reported outcomes related to clinician trust, reliance, and usability. The studies varied in imaging modality, clinical application, model architecture, and explanation-evaluation approach. However, limited assessment of faithfulness, robustness, usability, and clinician-related outcomes restricts conclusions about the clinical utility of the reported explanations. Previous reviews have addressed AI-based image analysis and XAI in dentistry, with partial overlap in scope (3–5, 19). The present review extends this literature by focusing specifically on dental and maxillofacial imaging and by systematically examining both the methods used to generate explanations and the rigor of their technical and clinician-centered evaluation.

The geographic concentration of studies in China and South Korea, also observed in bibliometric analyses of dental AI research (29), raises questions regarding the diversity of populations, institutions, imaging systems, acquisition protocols, and clinical workflows represented in the evidence base. For XAI specifically, this issue extends beyond model generalizability; explanations that appear meaningful in one dataset or clinical setting may not remain stable or clinically informative in another. Evaluation across geographically and technically diverse datasets is therefore important for determining whether explanation behavior generalizes alongside predictive performance.

Panoramic radiography was the most frequently represented modality, whereas intraoral radiography accounted for a smaller proportion of studies. Although the reasons for this distribution cannot be determined from the available evidence, the concentration of XAI research within particular imaging modalities means that evidence regarding explanation performance should not automatically be generalized across dental imaging techniques. Differences in image dimensionality, field of view, anatomical complexity, and acquisition characteristics may influence both model behavior and the explanations generated. Future studies should therefore evaluate predictive performance and explanation characteristics using independent data across different imaging systems and patient populations.

CAM-family methods, particularly Grad-CAM, dominated the XAI approaches used in the included studies. This methodological concentration is important because repeated use of a particular explanation method does not establish its superiority or suitability for dental imaging. Although several studies applied more than one XAI method, prespecified comparisons based on faithfulness, robustness, and clinician-centered outcomes were lacking. Consequently, the relative value of Grad-CAM and alternative approaches such as LIME (9), SHAP (10), and attention-based visualization remains uncertain. This is particularly relevant because visually intuitive explanations may appear clinically convincing without necessarily representing the features that actually influenced model predictions. Similar concerns have been raised in medical imaging, where technical success of CAD systems has not always translated into clinical benefit (30).

Local explanations were also considerably more common than approaches incorporating global explanations. Local explanations can help clinicians understand individual predictions, whereas global explanations provide information about broader model behavior and feature relationships. The predominance of local approaches may therefore reflect an emphasis on case-level visual interpretability rather than understanding how models behave systematically across populations. However, neither local nor global explanations inherently establish faithfulness or clinical usefulness, and the appropriate level of explanation is likely to depend on the intended user, model task, and clinical context.

Rigorous XAI evaluation should distinguish whether an explanation is clinically plausible from whether it is faithful to the model's predictive behavior (6). This distinction is important because agreement between a heatmap and an expected anatomical region may reassure a clinician while providing little evidence that the highlighted region actually drove the prediction. The absence of formal faithfulness assessment therefore represents a fundamental gap; without such testing, plausible explanations risk being interpreted as evidence of model reasoning when they may only provide visually convincing representations. Explanation evaluation should consequently combine complementary measures of faithfulness, localization, robustness, comprehensibility, and clinical relevance rather than relying predominantly on visual inspection.

A previous scoping review of AI-based image analysis in dental clinical decision-making similarly identified limited evidence from prospective studies assessing effects on clinician decisions, workflows, or patient outcomes (4). The present findings extend this concern to the evaluation of explanations themselves, suggesting that increasing the interpretability of an AI system does not necessarily establish its clinical value. This issue should also be considered alongside the high frequency of overall high-risk-of-bias judgments, although risk of bias and explanation-evaluation quality represent distinct methodological concerns and should be interpreted separately.

External validation was uncommon, which further limits the interpretation of XAI findings. For explainable AI, external validation has a dual importance: it should establish not only whether predictive performance is maintained in new populations and imaging environments, but also whether the explanations remain consistent and clinically meaningful. A model could potentially maintain acceptable predictive accuracy while relying on different features in a new setting. External evaluation of explanation behavior should therefore accompany conventional assessment of model performance.

Clinician-centered outcomes were also infrequently evaluated. This creates an important gap between technical explainability and clinically useful explainability. Trust, usability, acceptance, and reliance are distinct constructs, and a technically interpretable model cannot be assumed to improve any of them. Perceived usefulness and ease of use are established determinants of technology acceptance (31), whereas trust and reliance may additionally depend on system performance, uncertainty, explanation quality, prior experience, and clinical context. Future studies should therefore determine whether explanations improve clinician understanding, diagnostic performance, error detection, decision quality, or appropriately calibrated reliance rather than assuming that providing an explanation itself improves clinical acceptance.

Similarly, reader studies and expert review provide different levels of evidence. Expert appraisal can establish whether an explanation appears anatomically or clinically plausible, whereas structured reader studies can examine whether providing the explanation actually changes human performance or decision-making. This distinction is important because an explanation judged plausible by an expert may still have no beneficial effect on diagnostic decisions and could potentially encourage inappropriate reliance. Recent ethical and governance guidance for healthcare AI similarly emphasizes transparent reporting, risk management, and clinical evaluation alongside technical performance (15).

Quantitative localization and qualitative visual assessment provide useful information about explanation characteristics, but they represent intermediate evaluation outcomes rather than evidence of clinical benefit. Progress toward clinically meaningful XAI will therefore require moving beyond demonstrating that an explanation looks reasonable or overlaps with an expected anatomical region toward establishing that it is faithful, reproducible, and beneficial to clinical decision-making.

Several limitations should be acknowledged. Restricting the search to six databases and English-language articles may have missed relevant studies published elsewhere or in other languages. Potential publication bias should also be considered, as studies reporting more favorable or conclusive XAI findings may have been more likely to be published. Coding decisions required subjective interpretation, especially when studies used ambiguous terms or did not clearly define their XAI methods; the pilot coding and independent review mitigated this but did not eliminate it entirely. Classification according to the principal clinical application may not fully capture studies addressing multiple clinical applications. The near-total absence of external validation limits the generalizability of the findings reported in this review. More importantly, most included studies did not assess actual clinical outcomes; instead, they reported technical metrics or visual plausibility of explanations but rarely examined whether XAI outputs changed diagnostic decisions or improved patient care. A quantitative synthesis or meta-analysis was not performed because of substantial heterogeneity across the included studies in imaging modalities, clinical applications, AI models, XAI methods, and evaluation approaches. Additionally, the quality of XAI explanation evaluation could not be assessed using a dedicated standardized instrument, highlighting the need for validated XAI-specific evaluation and reporting frameworks. Finally, the lack of standardized reporting for XAI research in dentistry meant that many papers omitted methodological details, which limited the depth of synthesis.

Recent studies in dental and maxillofacial imaging have used several XAI approaches, although CAM-family methods predominated. However, confidence in the current evidence is limited by the high proportion of studies judged to have a high overall risk of bias, absence of formal explanation faithfulness assessment, and minimal evaluation of clinician trust, reliance, and usability. Future research should combine technically rigorous assessment of faithfulness, robustness, and localization with clinician-centered evaluation to determine whether explanations improve understanding, diagnostic performance, decision quality, and appropriately calibrated reliance.

## Data Availability

The original contributions presented in the study are included in the article/Supplementary Material, further inquiries can be directed to the corresponding author.
