# Supplementary material for: Explainable artificial intelligence in dental imaging: a systematic review of interpretability and the current state of trust evidence
Source: Front Dent Med. 2026 Sep 11;7:1948676. doi: 10.3389/fdmed.2026.1948676 (PMC13612324; doi:10.3389/fdmed.2026.1948676)
Supplement: Supplementary file 1 [file Table1.docx]

**Table S1.** Full search syntax adapted for each electronic database

| **Source** | **Full search syntax** |
| --- | --- |
| PubMed (MEDLINE) | ("Dental Imaging"[Mesh] OR "Radiography, Dental"[Mesh] OR "Radiography, Panoramic"[Mesh] OR "Cone-Beam Computed Tomography"[Mesh] OR "Cephalometry"[Mesh] OR "dental imaging"[TIAB] OR "dental radiograph*"[TIAB] OR panoramic[TIAB] OR periapical[TIAB] OR bitewing[TIAB] OR CBCT[TIAB] OR cephalometric[TIAB] OR "intraoral image*"[TIAB] OR "oral photograph*"[TIAB]) AND ("Artificial Intelligence"[Mesh] OR "Machine Learning"[Mesh] OR "Deep Learning"[Mesh] OR "Neural Networks, Computer"[Mesh] OR "artificial intelligence"[TIAB] OR "machine learning"[TIAB] OR "deep learning"[TIAB] OR CNN[TIAB] OR transformer[TIAB] OR "neural network*"[TIAB]) AND ("Explainable AI"[TIAB] OR "explainable artificial intelligence"[TIAB] OR interpretable[TIAB] OR interpretability[TIAB] OR explainable[TIAB] OR explainability[TIAB] OR "Grad-CAM"[TIAB] OR SHAP[TIAB] OR LIME[TIAB] OR "saliency map*"[TIAB] OR "attention map*"[TIAB] OR occlusion[TIAB] OR counterfactual[TIAB]) |
| Scopus | TITLE-ABS-KEY(("dental imaging" OR "dental radiograph*" OR panoramic OR periapical OR bitewing OR CBCT OR cephalometric OR "intraoral image*" OR "oral photograph*") AND ("artificial intelligence" OR "machine learning" OR "deep learning" OR CNN OR transformer OR "neural network*") AND ("explainable artificial intelligence" OR "explainable AI" OR interpretable OR interpretability OR explainable OR explainability OR "Grad-CAM" OR SHAP OR LIME OR "saliency map*" OR "attention map*" OR occlusion OR counterfactual)) |
| Web of Science | TS=((("dental imaging" OR "dental radiograph*" OR panoramic OR periapical OR bitewing OR CBCT OR cephalometric OR "intraoral image*" OR "oral photograph*")) AND (("artificial intelligence" OR "machine learning" OR "deep learning" OR CNN OR transformer OR "neural network*")) AND (("explainable artificial intelligence" OR "explainable AI" OR interpretable OR interpretability OR explainable OR explainability OR "Grad-CAM" OR SHAP OR LIME OR "saliency map*" OR "attention map*" OR occlusion OR counterfactual))) |
| IEEE Xplore | ("dental imaging" OR "dental radiograph" OR panoramic OR periapical OR bitewing OR CBCT OR cephalometric OR "intraoral image" OR "oral photograph") AND ("artificial intelligence" OR "machine learning" OR "deep learning" OR CNN OR transformer OR "neural network") AND ("explainable" OR "interpretable" OR "interpretability" OR "explainability" OR "Grad-CAM" OR SHAP OR LIME OR "saliency map" OR "attention map" OR occlusion OR counterfactual) AND ("dental" OR "tooth" OR "teeth" OR "oral" OR "maxillofacial" OR "jaw") |
| ScienceDirect | ("dental imaging" OR "dental radiograph" OR panoramic OR periapical OR bitewing OR CBCT OR cephalometric OR "intraoral image" OR "oral photograph") AND ("artificial intelligence" OR "machine learning" OR "deep learning" OR CNN OR transformer OR "neural network") AND ("explainable" OR "interpretable" OR "interpretability" OR "explainability" OR "Grad-CAM" OR SHAP OR LIME OR "saliency map" OR "attention map" OR occlusion OR counterfactual) |
| SpringerLink | Box 1 (All of these words): "dental imaging" OR "dental radiograph" OR panoramic OR periapical OR bitewing OR CBCT OR cephalometric  Box 2 (All of these words): "artificial intelligence" OR "machine learning" OR "deep learning" OR CNN OR transformer  Box 3 (All of these words): explainable OR interpretable OR SHAP OR LIME OR "saliency map" OR "attention map" |
