# Supplementary material for: Explainable artificial intelligence in dental imaging: a systematic review of interpretability and the current state of trust evidence
Source: Front Dent Med. 2026 Sep 11;7:1948676. doi: 10.3389/fdmed.2026.1948676 (PMC13612324; doi:10.3389/fdmed.2026.1948676)
Supplement: Supplementary file 2 [file Table2.docx]

**Table S2.** Operational definitions for data extraction variables

|  | **Variable** | **Operational definition** | **Coding categories** | **Coding definition** |
| --- | --- | --- | --- | --- |
| 1 | Bibliographic information | Basic citation details of the included study | Author | The surname of the first author of the study |
|  |  |  | Year | The publication year of the study as a four-digit number |
|  |  |  | Country | The country of the study, dataset, or main institution. Prefer the country where the data/images were collected. If not reported, use the first author affiliation country |
| 2 | Dental imaging modality | The type of dental radiographic or photographic imaging used as input to the AI model | Panoramic radiograph | A two-dimensional dental X-ray that captures the entire mouth in a single image, including teeth, jaws, and surrounding structures |
|  |  |  | Intraoral radiograph | A dental X-ray taken with the image receptor placed inside the oral cavity. This category includes periapical radiographs (showing the entire tooth from crown to root tip, including surrounding bone and apex) and bitewing radiographs (showing the crowns of upper and lower teeth simultaneously, used primarily for detecting interproximal caries) |
|  |  |  | CBCT | A three-dimensional imaging technique that produces volumetric data of the maxillofacial region using a cone-shaped X-ray beam |
|  |  |  | Cephalometric radiograph | A standardized lateral or frontal radiograph of the skull used for orthodontic assessment and treatment planning |
|  |  |  | Intraoral photograph | A clinical photograph taken inside the oral cavity using a dental camera |
|  |  |  | Oral photograph | A clinical photograph of the oral cavity, lips, or perioral region |
|  |  |  | Extraoral photograph | A clinical photograph taken outside the oral cavity, such as facial or profile photographs |
|  |  |  | MRI | A medical imaging technique using magnetic fields to produce detailed images of soft tissues in the maxillofacial region |
|  |  |  | 3D facial scan | A three-dimensional surface scan of the face used for orthodontic or maxillofacial assessment |
|  |  |  | Other | Any imaging modality not listed above (specify) |
| 3 | Dental specialty and clinical application | The dental specialty area or the clinical task the AI model was designed to address | Dental specialty/application | Oral Radiology, Endodontics, Orthodontics, Periodontics, Oral Surgery, Prosthodontics, Pediatric Dentistry, General Dentistry, Forensic Dentistry, Orofacial Pain, OMFR/OMFS |
| 4 | XAI method used and generated outputs | The specific XAI technique employed and the type of explanation output it produced | XAI method | Grad-CAM, Grad-CAM++, CAM, Score-CAM, SHAP, LIME, Saliency maps, Attention maps, Occlusion sensitivity, Counterfactual explanations, Integrated Gradients, LRP, EigenCAM, FullGrad, SmoothGrad, Visual analytics, Rule-based explanation, Feature importance, Attention rollout, CAMERAS, Other (specify) |
|  |  |  | XAI output | Heatmap, Saliency map, Attention map, Feature importance plot, Bounding box, Segmentation mask, Text explanation, Visual dashboard, Nomogram, 3D heatmap, Overlay map, Patch-weight map, Activation map, Superpixel map, Decision line, Confidence interval, Color map, Reconstructed image, Uncertainty interval, Other (specify) |
| 5 | Type of explanation | Classification of the explanation along two dimensions: (a) scope and (b) model dependency | Scope: Local | The explanation pertains to a single specific prediction for one input instance (e.g., a heatmap showing which pixels influenced the model's decision for a particular radiograph). Explanations are generated and presented on a per-image or per-case basis |
|  |  |  | Scope: Global | The explanation describes the overall behavior or logic of the model across all inputs (e.g., feature importance rankings derived from the entire dataset, or a decision tree showing the model's general decision rules). Explanations are model-level rather than instance-specific |
|  |  |  | Scope: Both | The study provides both local (per-instance) and global (model-level) explanations |
|  |  |  | Scope: Unclear | The study does not clearly specify whether explanations are local or global, or it cannot be determined from the reported information |
|  |  |  | Model dependency: Post-hoc | The explanation is generated after model training using a separate explainability technique applied to an already-trained black-box model (e.g., Grad-CAM, SHAP, LIME). The explanation is not derived from the model's internal structure but from a secondary method applied post-training |
|  |  |  | Model dependency: Intrinsic | The model is designed to be transparent by nature and does not require a separate post-hoc explanation method. The explanation is derived directly from the model's internal structure (e.g., decision trees, attention maps that are part of the model architecture, rule-based models, linear models) |
|  |  |  | Model dependency: Both | The study uses both post-hoc explainability techniques and intrinsically interpretable models, or provides both types of explanations |
|  |  |  | Model dependency: Unclear | The study does not clearly specify whether the explanation method is post-hoc or intrinsic, or it cannot be determined from the reported information |
| 6 | Evaluation approach for generated explanations | The method(s) used to assess the quality, validity, or usefulness of the XAI outputs | Evaluation type | The primary method used to assess or validate the XAI outputs. Categories include: Qualitative (visual inspection without quantitative metrics), Expert (clinicians or domain experts qualitatively assessed clinical plausibility), Localization (quantitative spatial alignment with ground-truth, e.g., IoU, Dice), Quantitative (general quantitative assessment such as ablation studies), Feature-importance (assessment based on feature importance plots without additional validation), Questionnaire (structured surveys), Usability (focus on practical usability, workflow integration, user satisfaction), Ablation (removing or modifying components to assess impact) |
|  |  |  | Expert review | Whether a dentist, oral radiologist, or clinical expert reviewed the explanation. Coded as Yes if clinical experts reviewed heatmaps, explanation maps, or AI outputs; No if no expert review is reported |
|  |  |  | Reader study | A formal study in which clinicians or human readers subjectively assess the quality, usefulness, clarity, or clinical plausibility of the XAI outputs (e.g., rating heatmaps on a Likert scale, ranking explanation quality, or providing feedback through interviews or surveys). Dentists/radiologists interpreted cases under controlled conditions and their performance was compared (e.g., before vs after AI/XAI assistance). Coded as Yes if conducted; No if not conducted |
| 7 | Outcomes related to clinical trust, usability, interpretability, clinical confidence, decision support, or clinical adoption | Reported outcomes assessing the clinical relevance, acceptance, or practical integration of the AI-XAI system | Trust measured? | Whether trust, confidence, or acceptance of the AI/XAI system was measured. Coded as Yes if questionnaires, Likert scales, confidence scores, or trust surveys were used; No if not measured or reported |
|  |  |  | Usability measured? | Whether ease of use or workflow usability was measured. Coded as Yes if system usability, user satisfaction, workflow integration, or user experience was evaluated; No if not measured or reported |
|  |  |  | Faithfulness tested? | Whether the study tested whether the explanation truly reflects the model decision process. Coded as Yes if occlusion, ablation, deletion/insertion tests, sanity checks, or similar methods were used; No if not tested |
|  |  |  | Localization checked? | Whether the explanation was compared with the true disease/lesion location. Coded as Yes if heatmaps or highlighted regions were compared with expert annotations, masks, bounding boxes, or lesion areas; No if not checked |
| 8 | External validation status | Whether the trained model was tested on an independent external dataset not used for training or internal validation | Yes | The model was tested on a dataset that was not used for training, tuning, or internal cross-validation, and which originated from a different source, center, institution, country, or time period. The test set from the same dataset is not considered external validation |
|  |  |  | No | External validation was not performed. The model was validated only using internal data (e.g., split from the same dataset, cross-validation) |
|  |  |  | Unclear | External validation was not clearly reported or could not be determined from the information provided |

CBCT: cone-beam computed tomography, MRI: magnetic resonance imaging, OMFR/OMFS: oral and maxillofacial radiology/surgery, XAI: explainable AI, CAM: class activation mapping, Grad-CAM: gradient-weighted class activation mapping, SHAP: Shapley additive explanations, LIME: local interpretable model-agnostic explanations, LRP: layer-wise relevance propagation, EigenCAM: eigen decomposition-based class activation mapping, FullGrad: full-gradient representation for neural network visualization, CAMERAS: class activation mapping for three-dimensional images.
