# Supplementary material for: Explainable artificial intelligence in dental imaging: a systematic review of interpretability and the current state of trust evidence
Source: Front Dent Med. 2026 Sep 11;7:1948676. doi: 10.3389/fdmed.2026.1948676 (PMC13612324; doi:10.3389/fdmed.2026.1948676)
Supplement: Supplementary file 3 [file Table3.docx]

**Table S3.** The complete extraction table with all variables

|  | **Bibliographic information** | | **Dataset** | | | **Explainable AI** | | | | **Evaluating the interpretation** | | | | | | | | **External validation** |
| --- | --- | --- | --- | --- | --- | --- | --- | --- | --- | --- | --- | --- | --- | --- | --- | --- | --- | --- |
| **#** | **Author & Year** | **Country** | **Imaging modality** | **Number of images** | **Dental application** | **XAI method** | **Scope** | **Model dependency** | **Output** | **Evaluation type** | **Expert review** | | **Reader study? yes/no** | **Trust measured? yes/no** | **Usability measured? yes/no** | **Faithfulness tested? yes/no** | **Localization checked? yes/no** | **External validation? yes/no** |
| 1 | Asiri, 2025^[23]^ | Saudi Arabia | Intraoral photographs | 2,000 | Dental caries | Grad-CAM | Local | Post-hoc | Heatmap | Qualitative | No | No | | No | No | No | Yes | No |
| 2 | Sreeram, 2025^[24]^ | India | Panoramic radiographs | NR | OMFR/ OMFS | Grad-CAM | Local | Post-hoc | Heatmap; Segmentation mask | Qualitative | No | No | | No | No | No | Yes | No |
| 3 | Çakmak, 2026^[25]^ | Turkey | Panoramic radiographs | 1,252 | Endodontics | Grad-CAM | Local | Post-hoc | Heatmap; Overlay | Qualitative | No | No | | No | No | No | Yes | No |
| 4 | Ovuz, 2026^[26]^ | Turkey | CBCT | 1,400 | OMFR/ OMFS | Grad-CAM | Local | Post-hoc | Heatmap | Qualitative | No | No | | No | No | No | Yes | No |
| 5 | Palaz, 2026^[27]^ | Turkey | Panoramic radiographs | 600 | Pediatric dentistry | Grad-CAM; SmoothGrad; Occlusion sensitivity | Local | Post-hoc | Heatmap; Saliency map; Occlusion map | Expert | Yes | No | | No | No | No | Yes | No |
| 6 | Ali, 2024^[28]^ | Indonesia | Intraoral radiographs | 862 | Forensic dentistry | SHAP | Global | Post-hoc | Feature importance plot | Feature-importance | No | No | | No | No | No | No | No |
| 7 | Rana, 2026^[29]^ | India | Panoramic radiographs | 232 | OMFR/ OMFS | Grad-CAM / SHAP /Saliency maps | Local | Post-hoc | Heatmap; Saliency map | Qualitative | No | No | | No | No | No | Yes | No |
| 8 | Oztekin, 2023^[30]^ | Turkey | Panoramic radiographs | 2,200 | Dental caries | Grad-CAM | Local | Post-hoc | Heatmap | Localization | Yes | No | | No | No | No | Yes | No |
| 9 | Bergner, 2022^[31]^ | Brazil | Intraoral radiographs | 38,174 | Dental caries | Saliency maps; Grad-CAM; Occlusion sensitivity | Local | Intrinsic | Heatmap; Patch-weight map | Localization | Yes | No | | No | No | No | Yes | No |
| 10 | Pishghadam, 2025^[32]^ | Iran | CBCT | 2,426 | Forensic dentistry | Grad-CAM; Attention maps | Local | Both | Heatmap; Attention map | Expert | Yes | No | | No | No | No | Yes | No |
| 11 | Cai, 2023^[33]^ | China | 3D facial scans | 1,044 | Orthodontics | Grad-CAM | Local | Post-hoc | Heatmap; Regional weight map | Quantitative | Yes | No | | No | No | No | Yes | No |
| 12 | Kim, 2025^[34]^ | South Korea | Intraoral photographs | 17,946 | Dental caries | Grad-CAM | Local | Post-hoc | Heatmap; Mask overlay | Localization | Yes | No | | No | No | No | Yes | No |
| 13 | Ashi, 2026^[35]^ | Saudi Arabia | Intraoral photographs | 2,000 | Dental caries | Grad-CAM | Local | Post-hoc | Heatmap | Qualitative | No | No | | No | No | No | Yes | No |
| 14 | Qi, 2026^[36]^ | China | CBCT | 2,148 | Dental caries | CAM | Local | Post-hoc | CAM overlay | Qualitative | No | No | | No | No | No | Yes | No |
| 15 | Asghar, 2025^[37]^ | Unknown (public dataset) | Intraoral photographs | 2,330 | Dental caries | Grad-CAM | Local | Post-hoc | Heatmap | Qualitative | No | No | | No | No | No | Yes | No |
| 16 | Jung, 2026^[38]^ | South Korea | Panoramic radiographs | 2,710 | Orofacial pain | Grad-CAM | Local | Both | Heatmap; Attention map | Ablation | No | No | | No | No | No | Yes | Yes |
| 17 | Zhang, 2025^[39]^ | China | Panoramic radiographs | 1,568 | Periodontology | Grad-CAM | Local | Post-hoc | Heatmap; Attention map | Qualitative | No | No | | No | No | No | Yes | No |
| 18 | Hayashi-Sakai, 2025^[40]^ | Japan | Panoramic radiographs | 628 | Pediatric dentistry | SHAP | Local | Post-hoc | SHAP heatmap | Qualitative | No | No | | No | No | No | Yes | No |
| 19 | Xu, 2025^[41]^ | China | CBCT | 2,146 | Orthodontics | Grad-CAM | Local | Post-hoc | Heatmap | Expert | Yes | Yes | | No | No | No | Yes | No |
| 20 | Ong, 2024^[42]^ | South Korea | Panoramic radiographs | 5,133 | Forensic dentistry | Grad-CAM | Local | Post-hoc | Heatmap | Qualitative | No | No | | No | No | No | Yes | No |
| 21 | Liu, 2024^[43]^ | China | Intraoral radiographs | 4,278 | Dental caries | Grad-CAM | Local | Post-hoc | Heatmap | Expert | Yes | Yes | | No | No | No | Yes | No |
| 22 | Vivel-Couso, 2026^[44]^ | Spain | Panoramic radiographs | 125 | Forensic dentistry | Grad-CAM | Local | Both | Heatmap; Text report | Questionnaire | Yes | Yes | | Yes | Yes | No | Yes | No |
| 23 | Barzas, 2025^[45]^ | Uruguay | CBCT | 20 | OMFR/ OMFS | Grad-CAM++ | Local | Post-hoc | Heatmap | Qualitative | No | No | | No | No | No | Yes | No |
| 24 | Chen, 2024^[46]^ | China | CBCT | 2,713 | Dental caries | LIME | Local | Post-hoc | Superpixel map; Segmentation mask | Expert | Yes | No | | No | No | No | Yes | No |
| 25 | Duru, 2026^[47]^ | Turkey | Panoramic radiographs | 1,222 | Forensic dentistry | Integrated gradients | Local | Post-hoc | Heatmap | Expert | Yes | No | | No | No | No | Yes | No |
| 26 | Inani, 2024^[48]^ | India | Intraoral photographs | 104 | Dental caries | Grad-CAM | Local | Post-hoc | Heatmap | Qualitative | No | No | | No | No | No | Yes | No |
| 27 | Parathanath, 2025^[49]^ | India | Intraoral radiographs | 1,103 | Dental caries | Attention maps | Unclear | Intrinsic | Attention-weighted feature map | Ablation | No | No | | No | No | No | No | No |
| 28 | Tahmasbi, 2026^[50]^ | Iran | Extraoral photographs | 524 | Orthodontics | Saliency maps | Local | Post-hoc | Saliency map | Qualitative | No | No | | No | No | No | Yes | No |
| 29 | Zhu, 2025^[51]^ | China | Panoramic radiographs | 521 | OMFR/ OMFS | Visual analytics | Both | Post-hoc | Visual dashboard | Usability | Yes | No | | No | Yes | No | Yes | No |
| 30 | Gwak, 2024^[52]^ | South Korea | Panoramic radiographs | 716 | OMFR/ OMFS | Grad-CAM | Local | Post-hoc | Binarized attention map | Localization | Yes | No | | No | No | No | Yes | No |
| 31 | Arian, 2023^[53]^ | Unknown (public dataset) | Panoramic radiographs | 706 | Forensic dentistry | FullGrad | Local | Post-hoc | Heatmap | Qualitative | No | No | | No | No | No | Yes | No |
| 32 | Wen, 2024^[54]^ | China | Intraoral photographs | 826 | Periodontology | Grad-CAM++ | Local | Post-hoc | Heatmap; Contour map; Feature plot | Localization | No | No | | No | No | No | Yes | No |
| 33 | Chen, 2023^[55]^ | China | Panoramic radiographs | 1,100 | Dental caries | Rule-based visual explanation | Local | Intrinsic | Segmentation mask; Decision line | Quantitative | No | No | | No | No | No | Yes | No |
| 34 | Kang, 2024^[56]^ | South Korea | Panoramic radiographs | 565 | OMFR/ OMFS | Attention maps | Both | Intrinsic | Attention map; Segmentation map | Localization | Yes | Yes | | No | No | No | Yes | No |
| 35 | Motmaen, 2024^[57]^ | Germany | Panoramic radiographs | 1,184 | OMFR/ OMFS | CAMERAS class activation mapping | Local | Post-hoc | Activation map | Expert | Yes | Yes | | No | No | No | Yes | No |
| 36 | Buyukcakir, 2025^[58]^ | Belgium | CBCT | 528 | Forensic dentistry | Attention rollout | Both | Intrinsic | 3D attention map | Qualitative | No | No | | No | No | No | Yes | No |
| 37 | Lee, 2024^[59]^ | South Korea | MRI | 2,948 | Orofacial pain | Grad-CAM | Local | Post-hoc | Heatmap; Activation map | Quantitative | Yes | Yes | | No | No | No | Yes | No |
| 38 | Yeom, 2023^[60]^ | South Korea | Panoramic radiographs | 9,663 | Forensic dentistry | Attention rollout | Local | Post-hoc | Heatmap | Qualitative | No | No | | No | No | No | Yes | No |
| 39 | Chen, 2026^[61]^ | China | CBCT | 285 | OMFR/ OMFS | Grad-CAM | Both | Post-hoc | 3D heatmap; Nomogram | Quantitative | No | No | | No | No | No | Yes | No |
| 40 | Li, 2025^[62]^ | China | CBCT | NR | Orthodontics | Integrated Gradients; SHAP; LIME; Attention maps | Both | Both | 3D visual explanation | Expert | Yes | Yes | | No | No | No | No | No |
| 41 | Zhang, 2023^[63]^ | China | Cephalometric radiographs | 296 | Orthodontics | Grad-CAM | Local | Post-hoc | Heatmap | Qualitative | Yes | Yes | | No | No | No | Yes | No |
| 42 | Miranda, 2023^[64]^ | USA & Brazil | CBCT | 194 | Orthodontics | Grad-CAM; Attention maps | Local | Both | 3D surface heatmap | Expert | Yes | No | | No | No | No | Yes | No |
| 43 | Chung, 2025^[65]^ | China | Intraoral photographs | 653 | Orthodontics | Score-CAM | Local | Post-hoc | Heatmap | Expert | Yes | No | | No | No | No | Yes | No |
| 44 | Zhou, 2023^[66]^ | China | Intraoral photographs | 10,200 | Forensic dentistry | Grad-CAM | Local | Post-hoc | Heatmap | Qualitative | No | No | | No | No | No | Yes | No |
| 45 | Simsek, 2025^[67]^ | Turkey | Panoramic radiographs | 626 | Forensic dentistry | SHAP | Global | Post-hoc | Feature importance plot | Feature-importance | No | No | | No | No | No | No | No |
| 46 | Lee, 2022^[68]^ | South Korea | MRI | 2,520 | Orofacial pain | Grad-CAM | Local | Post-hoc | Heatmap; Activation map | Quantitative | Yes | Yes | | No | No | No | Yes | No |
| 47 | Kayadibi, 2025^[69]^ | Turkey | Panoramic radiographs | 973 | OMFR/ OMFS | EigenCAM | Local | Post-hoc | Heatmap; Bounding box | Usability | Yes | Yes | | No | Yes | No | Yes | No |
| 48 | Choi, 2023^[70]^ | South Korea | Intraoral radiographs | 402 | Endodontics | Class activation map (CAM) | Local | Post-hoc | CAM | Qualitative | Yes | Yes | | No | No | No | Yes | No |
| 49 | Moreira, 2026^[71]^ | Brazil | CBCT | 2,648 | Forensic dentistry | Grad-CAM | Local | Post-hoc | Heatmap | Qualitative | No | No | | No | No | No | Yes | No |
| 50 | Lee, 2024^[72]^ | South Korea | Panoramic radiographs; MRI | 55; 16 | Orofacial pain | Grad-CAM | Local | Post-hoc | Heatmap | Qualitative | No | No | | No | No | No | Yes | No |
| 51 | Pereira, 2025^[73]^ | Portugal | Panoramic radiographs | 1,050 | Forensic dentistry | Grad-CAM | Local | Post-hoc | Heatmap | Qualitative | No | No | | No | No | No | Yes | No |
| 52 | Buyukcakir, 2025^[74]^ | Belgium | Panoramic radiographs | 790 | Forensic dentistry | Attention maps; Visual analytics | Both | Both | Attention map; Reconstructed image | Qualitative | No | No | | No | No | No | Yes | No |
| 53 | Joo, 2023^[75]^ | South Korea | Panoramic radiographs | 2,950 | Forensic dentistry | Visual analytics | Both | Intrinsic | Virtual image; Color map | Quantitative | No | No | | No | No | No | Yes | No |
| 54 | Qiu, 2026^[76]^ | China | CBCT | 425 | OMFR/ OMFS | SHAP | Both | Post-hoc | Feature importance plot | Feature-importance | Yes | Yes | | No | No | No | No | No |
| 55 | Ragodos, 2022^[77]^ | International | Intraoral photographs | 38,486 | OMFR/ OMFS | Saliency maps | Local | Post-hoc | Saliency map | Qualitative | Yes | Yes | | No | No | No | Yes | No |
| 56 | Zhang, 2022^[78]^ | China | Cephalometric radiographs | 14,142 | Orthodontics | Grad-CAM | Both | Post-hoc | Heatmap | Quantitative | No | No | | No | No | No | Yes | No |
| 57 | Xu, 2024^[79]^ | China | CBCT | 2,146 | Orthodontics | Grad-CAM | Local | Post-hoc | Heatmap | Qualitative | No | No | | No | No | No | Yes | No |
| 58 | Kavousinejad, 2026^[80]^ | Iran | Intraoral photographs | 9,292 | Orthodontics | Grad-CAM; Attention maps | Local | Both | Heatmap; Overlay | Expert | Yes | Yes | | No | No | No | Yes | Yes |
| 59 | Luo, 2026^[81]^ | China | Panoramic radiographs | 7307 | Forensic dentistry | Grad-CAM; Attention maps | Local | Both | Heatmap; Uncertainty interval | Quantitative | Yes | Yes | | No | No | No | Yes | No |
| 60 | Sivari Resul, 2026^[82]^ | Turkey | Panoramic radiographs | 1,170 | Pediatric dentistry | Grad-CAM; Attention maps | Local | Both | Heatmap | Expert | Yes | Yes | | No | No | No | Yes | No |
| 61 | Khurshid, 2026^[83]^ | Bangladesh | Intraoral photographs | 2,000 | Dental caries | Grad-CAM | Local | Post-hoc | Heatmap | Qualitative | No | No | | No | No | No | Yes | No |

CBCT: cone-beam computed tomography, MRI: magnetic resonance imaging, OMFR/OMFS: oral and maxillofacial radiology/surgery, XAI: explainable AI, CAM: class activation mapping, Grad-CAM: gradient-weighted class activation mapping, SHAP: Shapley additive explanations, LIME: local interpretable model-agnostic explanations, LRP: layer-wise relevance propagation, EigenCAM: eigen decomposition-based class activation mapping, FullGrad: full-gradient representation for neural network visualization, CAMERAS: class activation mapping for three-dimensional images.
