# Supplementary material for: Explainable artificial intelligence in dental imaging: a systematic review of interpretability and the current state of trust evidence
Source: Front Dent Med. 2026 Sep 11;7:1948676. doi: 10.3389/fdmed.2026.1948676 (PMC13612324; doi:10.3389/fdmed.2026.1948676)
Supplement: Supplementary file 4 [file Table4.docx]

**Table S4.** Distribution of XAI outputs across included studies

| Output type | Output subtype | Frequency (n) | Percentage (%) |
| --- | --- | --- | --- |
| Heatmap-based Outputs | Heatmap | 40 | 46.5 |
|  | SHAP heatmap | 1 | 1.2 |
|  | CAM overlay/ CAM | 2 | 2.3 |
|  | 3D heatmap | 1 | 1.2 |
|  | 3D surface | 1 | 1.2 |
| Attention-based Outputs | Attention map | 5 | 5.8 |
|  | Attention-weighted | 1 | 1.2 |
|  | Binarized | 1 | 1.2 |
|  | 3D attention | 1 | 1.2 |
|  | Segmentation map | 1 | 1.2 |
| Gradient-based Outputs | Saliency map | 4 | 4.7 |
|  | Activation map | 3 | 3.5 |
| Segmentation-based Outputs | Segmentation mask | 3 | 3.5 |
|  | Superpixel map | 1 | 1.2 |
| Feature importance | Feature importance plot | 4 | 4.7 |
| Overlay/Mask | Overlay | 2 | 2.3 |
|  | Mask overlay | 1 | 1.2 |
| Map variants | Patch-weight | 1 | 1.2 |
|  | Regional weight | 1 | 1.2 |
|  | Contour | 1 | 1.2 |
|  | Occlusion | 1 | 1.2 |
|  | Color map | 1 | 1.2 |
| Other visual | Visual dashboard | 1 | 1.2 |
|  | Reconstructed image | 1 | 1.2 |
|  | Virtual image | 1 | 1.2 |
|  | 3D visual explanation | 1 | 1.2 |
|  | Bounding box | 1 | 1.2 |
|  | Decision line | 1 | 1.2 |
| Text/Structured | Text report | 1 | 1.2 |
|  | Nomogram | 1 | 1.2 |
|  | Uncertainty interval | 1 | 1.2 |
