# Supplementary material for: Explainable artificial intelligence in dental imaging: a systematic review of interpretability and the current state of trust evidence
Source: Front Dent Med. 2026 Sep 11;7:1948676. doi: 10.3389/fdmed.2026.1948676 (PMC13612324; doi:10.3389/fdmed.2026.1948676)
Supplement: Supplementary file 5 [file Table5.docx]

**Table S5.** Distribution of XAI evaluation characteristics across included studies

| **Characteristic** | **Category** | **Number of studies (n)** | **Percentage (%)** |
| --- | --- | --- | --- |
| Evaluation type | Qualitative | 27 | 44.3 |
|  | Expert | 12 | 19.7 |
|  | Quantitative | 8 | 13.1 |
|  | Localization | 6 | 9.8 |
|  | Feature-importance | 3 | 4.9 |
|  | Usability | 2 | 3.3 |
|  | Ablation | 2 | 3.3 |
|  | Questionnaire | 1 | 1.6 |
| Expert review performed | Yes | 28 | 45.9 |
|  | No | 33 | 54.1 |
| Reader study performed | Yes | 16 | 26.2 |
|  | No | 45 | 73.8 |
| Trust measured | Yes | 1 | 1.6 |
|  | No | 60 | 98.4 |
| Usability measured | Yes | 3 | 4.9 |
|  | No | 58 | 95.1 |
| Faithfulness tested | Yes | 0 | 0 |
|  | No | 61 | 100 |
| Localization checked | Yes | 56 | 91.8 |
|  | No | 5 | 8.2 |
